# Workplace bullying and tiredness at work: A cross‐lagged prospective study of causal directions and the moderating effects of a conflict management climate

**DOI:** 10.1002/1348-9585.12327

**Published:** 2022-04-06

**Authors:** Michael Rosander, Morten Birkeland Nielsen

**Affiliations:** ^1^ 4566 Department of Behavioural Sciences and Learning Linköping University Linköping Sweden; ^2^ National Institute of Occupational Health Oslo Norway; ^3^ Department of Psychosocial Science University of Bergen Bergen Norway

**Keywords:** conflict management climate, sleep problems, tiredness, workplace bullying

## Abstract

**Objectives:**

To prospectively investigate the reciprocal associations between tiredness at work (TAW) and exposure to bullying behaviors and to determine the role of conflict management climate (CMC) as a moderator of these associations.

**Methods:**

A two‐wave national probability sample of employees in Sweden (18 months between waves, 921 participated at both waves) measuring TAW, workplace bullying, and CMC. Structural equation modelling was used to test four hypotheses about the longitudinal associations between feeling tired at work and bullying, and CMC as a moderator for the two directions.

**Results:**

In the analyses of cross‐lagged effects, tiredness was significantly associated with an increase in subsequent bullying (β = 0.08, *P =* .01). Exposure to bullying was not associated with changes in tiredness. CMC moderated the association between tiredness and subsequent bullying (β = −0.13, 95% CI [−0.19, −0.08]), showing an increased risk of exposure to bullying behaviors following tiredness when CMC was low and decreased risk when CMC was high.

**Conclusions:**

TAW is a risk factor for subsequent bullying. Finding ways to help employees to reduce tiredness not only will help them perform better at work but also reduce the risk of them becoming targets of bullying. A strong CMC can act as a buffer if a tired person provoke aggression from co‐workers.

## 1 INTRODUCTION

Work and sleep are strongly interconnected.^1^ One the one hand, exposure to unmanageable stressors at the workplace can become an obstacle negatively affecting sleep and recovery. On the other hand, going to work when feeling tired will impair cognitive abilities and thereby reduce focus and performance.^2^ As for specific work stressors, recent meta‐analytic findings suggests that exposure to workplace bullying is a significant, but understudied, work‐related correlate of sleep problems.^3^ In the limited research literature that exist, bullying has been examined as both a potential cause^4^, ^5^ and consequence^6^, ^7^, ^8^ of sleep problems and by extension tiredness at work (TAW). Consequently, the previous literature points to a potential reciprocal relationship between exposure to workplace bullying and TAW, where work‐related stress and tiredness reinforce and enhance each other mutually as in a vicious circle. Further knowledge about how workplace bullying and TAW are related is therefore highly important, both regarding how tiredness may be reduced in workers as well as for how to maintain optimal functioning and performance at the workplace. To add to the current knowledge base, the overarching aim of this study was to examine bidirectional time‐lagged relationships between exposure to workplace bullying and TAW. As an effort to enhance our understanding of how to reduce the detrimental effects of workplace bullying, a second aim was to examine whether a conflict management climate (CMC) moderates the reciprocal associations between workplace bullying and TAW.

Tiredness is a form of weariness and refers to the state of wishing for sleep or rest. Tiredness is considered as a consequence of sleep deprivation, be it qualitative or quantitative and in previous research on the association between sleep and work, it has been assumed that sleep problems will lead to TAW.^1^ In some studies, total sleep deprivation has been in focus^2^, ^9^ and with that a clearer connection between sleep and tiredness. For other studies, focusing on different levels of sleep problems, the connection is probably not as definite as the level of TAW may be dependent on work characteristics, for example, a stimulating job. There are studies on actual TAW, for example DeArmond and Chen^10^ who found a significant association between TAW and safety behavior. They distinguished between two related aspects of tiredness—tiredness from a physiological perspective and a more subjective form of tiredness. The physiological tiredness is a function of the quality and quantity of sleep. The subjective tiredness not only is influenced by the physiological tiredness but may also be influenced by factors in the work environment. It is the subjective aspect of TAW, and its relation to workplace bullying that is in focus for the current study.

Workplace bullying refers to a systematic form of harassment where an employee, persistently and over a period of time, is exposed to negative acts from superiors and/or co‐workers and where the employee finds it difficult to defend against these actions due to a real or perceived power imbalance between target and perpetrator.^11^ Exposure to bullying behaviors may come in different forms and intensities, and if this kind for exposure becomes prolonged and systematic, it may subsequently be experienced as a form of victimization.^12^ In the present study, we investigated the first part of this process, that is, exposure to bullying behaviors at different intensity. Comparisons with other psychosocial exposures such as high workload, poor safety, and leadership show that bullying is one of the most detrimental predictors of mental health problems and reduced workability.^13^, ^14^ Consequently, it is also reasonable to expect that exposure to bullying increases the likelihood of sleep problems, including tiredness. Specifically, workplace bullying represents a direct threat to the personal integrity of those exposed, and persistent exposure is associated with worrying,^15^ rumination, and physical activation.^16^, ^17^ It is therefore not surprising that evidence points to bullying as predictor of subsequent sleep problems. In their meta‐analysis of the relation between workplace bullying and sleep problems, Nielsen and colleagues^3^ found that exposure to bullying behaviors was associated with a 62% increase in sleep problems across time. Thus, there is reason to believe that exposure to bullying behaviors is associated to subsequent TAW.

Hypothesis 1 Controlling for baseline tiredness, exposure to bullying behaviors at T1 is associated with increased TAW at T2.

Theoretically, there is also a potential reversed effect of TAW on bullying which could be understood as a consequence of perceived rule violations where a tired co‐worker may trigger aggression from others.^18^ These rule violations can be work‐related and/or related to expectations of social conduct at work. Specifically, TAW may lead to reduced performance and general functioning at work.^2^, ^9^, ^19^ This inhibited performance may provoke others and lead to negative reactions from peers and superiors that, if sustained over time, may be perceived as bullying. In a related manner, a tired co‐worker perceived not to fulfil his or her duties may be singled out as a scapegoat in times of turmoil and problems at work. Thus, we expect to find that TAW affects subsequent exposure to bullying behaviors.

Hypothesis 2 Controlling for baseline exposure to bullying behaviors, TAW at T1 is associated with increased exposure to bullying behaviors at T2.

Despite theoretical reasons for expecting tiredness to be risk factors for workplace bullying, a substantial limitation of existing research is that most studies have largely ignored the potential reversed effect of tiredness on later risk of workplace bullying.^3^ A simultaneous analysis of the reciprocal relationships has only been included in one study which focused on the association between sleep problems and bullying. Examining full‐panel cross‐lagged relations between bullying and insomnia with a 6‐month time‐lag in a national probability sample comprising 1149 Norwegian employees, Nielsen and colleagues^20^ found evidence for an effect of bullying on increase in insomnia over time, but not any reversed effects of insomnia on the risk of bullying. However, as a bullying case is assumed to take at least 6 months to develop,^12^ it can be questioned whether a 6‐month time‐lag is adequate with regard to detecting an effect between sleep problems and bullying. In a meta‐analysis Ford and colleagues^21^ found that the effects of work stressors become more pronounced over time. They also found similar effects for reversed causations. It is therefore necessary to examine this relation with longer time‐lags as well as focusing on a potential consequence of sleep problems—TAW.

Another limitation of existing research on sleep, tiredness and workplace bullying is that few studies have investigated moderating variables that determine when and for whom bullying and tiredness are related.^3^ There is strong support for the link between interpersonal conflicts and exposure to bullying behaviors,^22^ and leader initiatives to reduce conflicts may, therefore, also contribute to reduce the occurrence of bullying. Supporting this notion, Leymann^23^ claimed that, unless a managerial intervention takes place, bullying will continue to escalate until it reaches a final “expulsion stage” where the target is forced out of his or her job or current position. As for specific leadership initiatives, recent evidence suggest that an organization’s CMC could be especially beneficial with regard to reducing the occurrence of workplace bullying.^24^, ^25^, ^26^ CMC is defined as a common assessment or belief among employees that conflicts, if they arise, will be handled successfully by the management and leaders in the organization.^27^ A strong CMC should reduce the risk that exposure to bullying behaviors escalates, and thereby contributing to lessen the impact of workplace bullying on those targeted.^28^, ^29^ Thus, we hypothesis that CMC will buffer the negative consequences of exposure to bullying behaviors. The ability to manage conflicts seems to have an immediate effect reducing conflicts and thereby preventing escalation possibly leading to bullying, as well as a long‐term effect affecting the climate at a workplace reducing the risk of interpersonal conflicts altogether.^27^, ^28^

Hypothesis 3 Controlling for baseline tiredness, a strong CMC will reduce the magnitude of the association between exposure to bullying behaviors at T1 and tiredness at T2.

In addition to reducing the impact of bullying, a strong CMC may diminish the risks connected to escalation of bullying, for instance emanating from failures to live up to expectations at work or rule violations due to tiredness. It is thereby likely that a strong CMC will reduce the risk of exposure to bullying behaviors due to TAW.

Hypothesis 4 Controlling for baseline exposure to bullying behaviors, a strong CMC will reduce the magnitude of the association between tiredness at T1 and exposure to bullying behaviors at T2.

Extending previous research, this study examines reciprocal relationships between exposure to workplace bullying and TAW using a one‐and‐a‐half‐year time‐lag. In addition, to understand the factors that determine when workplace bullying and tiredness are related, we examine whether CMC moderates the reciprocal associations between workplace bullying and TAW.

## 2 MATERIALS AND METHODS

The study is based on a longitudinal national probability sample drawn from the whole Swedish workforce (a total of 3.3 million employees). Statistics Sweden, a government agency, was responsible for all aspects regarding sampling procedures. To be included, a respondent had to be employed at a workplace with at least ten employees and be between 18 and 65 years of age. The first wave was collected in the autumn of 2017 (*n* = 1853) and the second in the spring of 2019 (*n* = 1095), that is, a time‐lag of 18 months. Only those who responded to the first wave were invited to the second wave. A change of jobs may be related to exposure workplace bullying and is also likely to influence the effects of CMC. Consequently, 174 respondents that had changed jobs between T1 and T2 were excluded from the analyses. The project was approved by the Regional Ethical Review Board at Linköping University, Sweden. Protocol number: 2017/336‐32.

### 2.1 Measures

The Short Negative Acts Questionnaire–Revised SNAQ–R,^30^, ^31^ was used to assess exposure to bullying behaviors at work. The SNAQ–R contains nine items reflecting different negative acts with a 5‐point frequency response scale from never to daily during the past 6 months. The variable was calculated using mean scores. The internal consistency (Cronbach’s alpha) of SNAQ–R was .83 at T1 and .85 at T2.

TAW was measured using three items focusing on feeling tired at work. One of the items use a semantic differential scale in six steps from “I have felt tired, exhausted” to “I have felt alert, wakeful” taken from the Salutogenic Health Indicator Scale^32^ and asks the respondent to assess the last 4 weeks. The other two items were taken from the Psychosocial Work Environment Questionnaire (PSYWEQ)^33^: “During the workday I feel wakeful and alert” and “When the workday is over I feel wakeful and alert” assessing the current situation. Both use a 7‐point Likert scale ranging from “Strongly disagree” to “Strongly agree”. Together the three items measure TAW. The mean scores for T1 and T2 were calculated using standardized items as the scales used are different. Cronbach’s alpha for T1 was .80 and for T2 .80.

CMC is a measure about the confidence felt about a successful resolution of conflicts that arises at the workplace. It is a measure taken from the PSYWEQ comprising three items: “If there is a serious conflict, I trust it will be resolved in a good way,” “We are good at resolving conflicts,” and “The supervisor is good at resolving conflicts.” Responses were given on a 7‐point Likert scale ranging from “Strongly disagree” to “Strongly agree.” The variable was calculated using mean scores. Cronbach’s alpha for T1 was .88 and for T2 .86.

Statistics Sweden added demographic information to the data taken directly from the Swedish population register. Whether a respondent held a managerial position, had a fixed contract, and the time they had worked at their current workplace were collected through direct questions in the survey.

### 2.2 Statistical analyses

We used Stata 17 for all analyses. Structural equation modelling (SEM) and the maximum likelihood with missing values were used as the main analytic method. Model fit was determined using chi‐squared (χ^2^), root mean square error of approximation (RMSEA), comparative fit index (CFI), and Tucker–Lewis index (TLI). Values below 0.05 for the RMSEA and close to 0.95 for the CFI and TLI was used as indication of a good fit.^34^ The Akaike’s information criterion (AIC) was used to compare non‐nested models. We used standardized items to calculate the interaction variables and for the other the manifest variables in the SEMs. In all analyses we adjusted for sex, age, and managerial position. Sex and age were added as there are indications that both may be related to exposure to bullying behaviors.^35^, ^36^ Managerial position was added as such a position may have an influence on bullying based on being higher up in the hierarchy in an organization and bullying contains by definition elements of power differences.^12^

## 3 RESULTS

### 3.1 Descriptive findings

The sample comprised 58% women, 90% were born in Sweden, 55% were married, 14% had managerial position, and 97% had a fixed contract. The mean age of the participants was 50.1 years (SD = 9.8). They had worked at the current workplace on average 14.2 years (SD = 11.8). The majority (59%) had some form of university or college education, 1% had less than 9 years of schooling, 4% had only 9–10 years (compulsory school), and 35% had 11–12 years.

Means, standard deviations, and intercorrelations for all study variables are presented in Table 1.

**TABLE 1:** Means, standard deviations, and intercorrelations for the study variables

	Mean	SD	1	2	3	4	5	6	7	8
1. Sex	–	–	–							
2. Age	50.08	9.76	0.00	–						
3. Position	–	–	−0.10**	0.02	–					
4. SNAQ–R T1	1.20	0.34	−0.05	−0.11***	0.01	–				
5. SNAQ–R T2	1.19	0.33	−0.05	−0.11***	0.00	0.63***	–			
6. TAW T1	0	0.85	0.12***	−0.07*	−0.07*	0.27***	0.24***	–		
7. TAW T2	0	0.85	0.12***	−0.10**	−0.03	0.24***	0.27***	0.68***	–	
8. CMC T1	4.56	1.62	−0.04	0.00	0.13***	−0.35***	−0.33***	−0.39***	−0.30***	
9. CMC T2	4.62	1.60	−0.05	0.05	0.13***	−0.32***	−0.41***	−0.35***	−0.38***	0.63***

Abbreviation

CMC, conflict management climate; SNAQ–R, Short Negative Acts Questionnaire–Revised; TAW, tiredness at work. *Sex and Position are dichotomous variables*.

* *P < *.05, **P < .01, ***P < .001.

### 3.2 Testing the measures

To determine whether the indicators of workplace bullying, TAW, and CMC were empirically different, we followed a confirmatory approach that compared a one‐factor measurement model (i.e., all items loading on a single factor) with a three‐factor solution (i.e., three latent variables as described in the methods section). The results, testing at T1 and at T2, clearly showed a better fit for the three latent variables model. The fit statistics for one latent variable at T1 were χ^2^(90) = 2570.54, *P <* .001, CFI = 0.56, TLI = 0.49, RMSEA = 0.173 (90% CI = 0.167–0.179). For the three latent variables model they were χ^2^(87) = 445.18, *P <* .001, CFI = 0.94, TLI = 0.92, RMSEA = 0.067 (90% CI = 0.061–0.073). For T2, the results were similar. The fit statistics for one latent variable were χ^2^(90) = 2364.07, *P <* .001, CFI = 0.61, TLI = 0.54, RMSEA = 0.166 (90% CI = 0.160–0.172). For the three latent variables model they were χ^2^(87) = 474.24, *P <* .001, CFI = 0.93, TLI = 0.92, RMSEA = 0.070 (90% CI = 0.063–0.076).

### 3.3 Full panel comparisons

**FIGURE 1:**
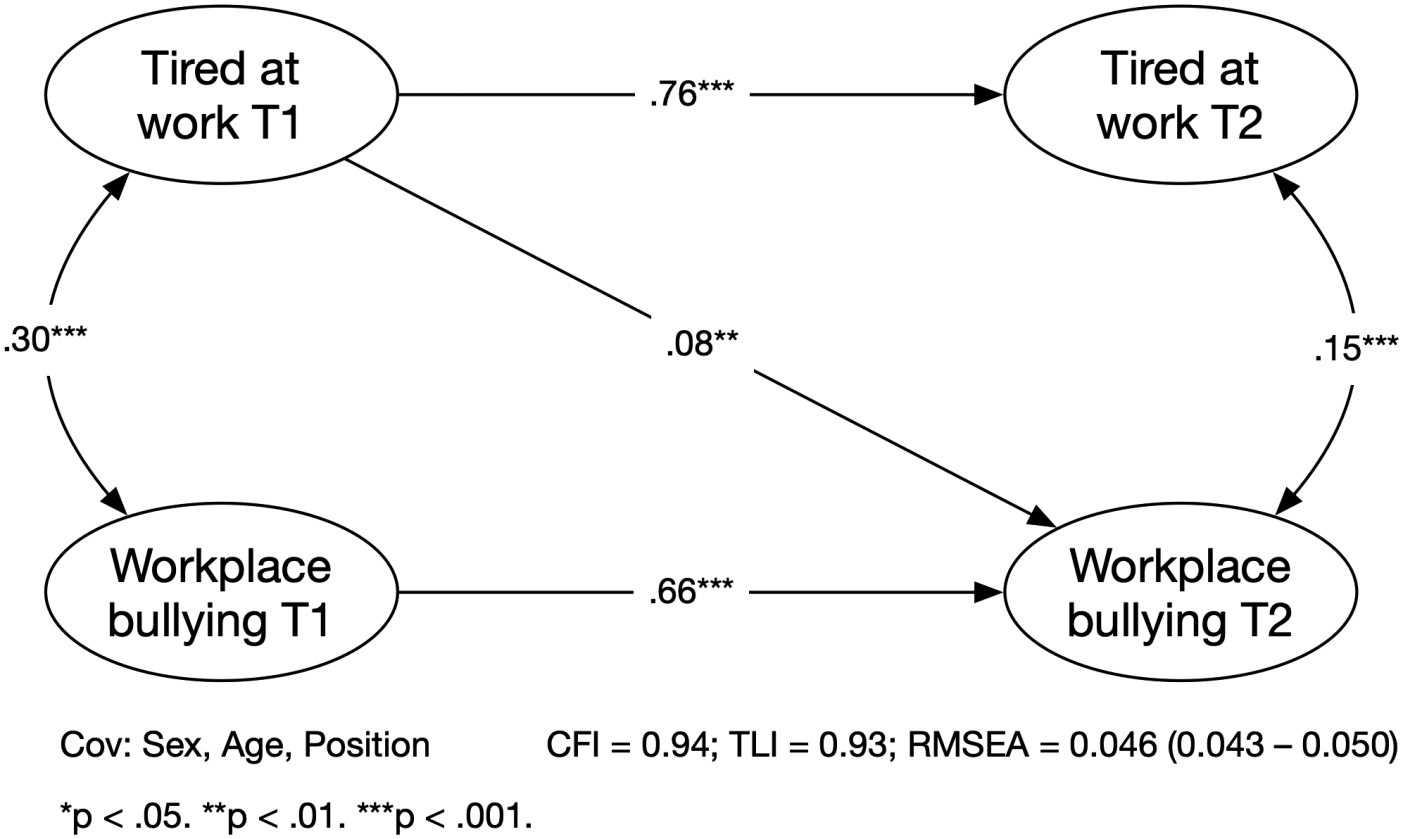
Associations between tiredness at work and exposure to bullying behaviors adjusted for age, gender, and leadership position (M3 model). Standardized coefficients

We tested and compared four models, a stability model, a forward and a reversed model, and a reciprocal model (see Table 2). All models were adjusted for sex, age, and leadership position. The reverse model (M3, Tired T1 → Bullying T2) had better fit to the data compared with the stability model (M1) and the forward model (M2, bullying T1 → Tired T2). The reciprocal model (M4) did not improve the fit compared with the M3 model. The only significant cross‐lagged association was TAW at baseline and exposure to bullying behaviors at follow‐up in the reversed model, β = 0.08, *P =* .010 (see Figure 1). For the forward model, the association between exposure to bullying behaviors at baseline and tiredness at follow‐up was not significant, β = 0.04, *P =* .194. Of the covariates, only sex showed a significant association to tired at work at follow‐up, β = 0.05, *P =* .046. All others did not show any significant association to either exposure to bullying behaviors or TAW at follow‐up. Hence, the data indicated that there was an effect of TAW on exposure to bullying behaviors, whereas no effect were found for exposure to bullying behaviors on TAW. Thus, the results only support Hypothesis 2, not Hypothesis 1.

**TABLE 2:** Results of four cross‐lagged structural regression models between workplace bullying and tiredness at work

		Test statistics					Model comparison	
		χ^2^	*df*	CFI	TLI	RMSEA (90% CI)	Comparison	(*df*) χ^2^
M1	Stability model	888.32***	297	0.94	0.93	0.047 (0.043–0.050)		
M2	Forward model (bullying T1 → tiredness T2)	886.64***	296	0.94	0.93	0.047 (0.043–0.050)	M2 vs. M1	(1) 1.68 ns
M3	Reversed model (tiredness T1 → bullying T2)	881.77***	296	0.94	0.93	0.046 (0.043–0.050)	M3 vs. M1 M3 vs. M2	(1) 6.55* (–) 4.87*
M4	Reciprocal model (bullying T1 → tiredness T2 and tiredness T1 → bullying T2)	880.19***	295	0.94	0.93	0.046 (0.043–0.050)	M4 vs. M1 M4 vs. M2 M4 vs. M3	(2) 8.13* (1) 6.45* (1) 1.58 ns

Abbreviation

CI, confidence interval; CFI, comparative fit index; ns, not significant; RMSEA, root mean square error of approximation; TLI, Tucker–Lewis index.

* *P* < .05

** *P* < .01

*** *P* < .001.

### 3.4 Conflict management climate as a moderator

**FIGURE 2:**
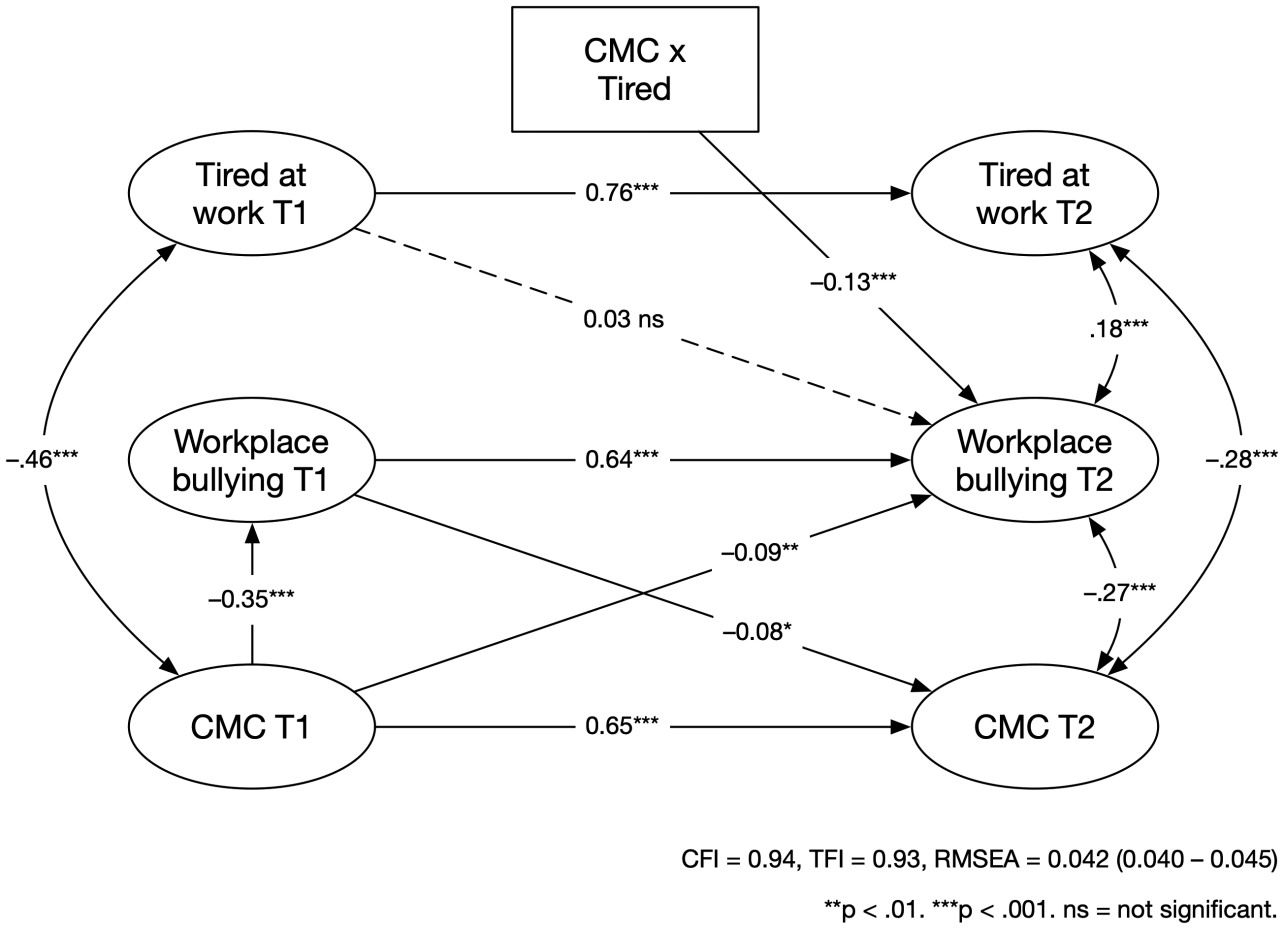
Associations between tiredness at work and exposure to bullying behaviors including the conflict management climate as a moderator adjusted for age, gender, and leadership position. Standardized coefficients

**FIGURE 3:**
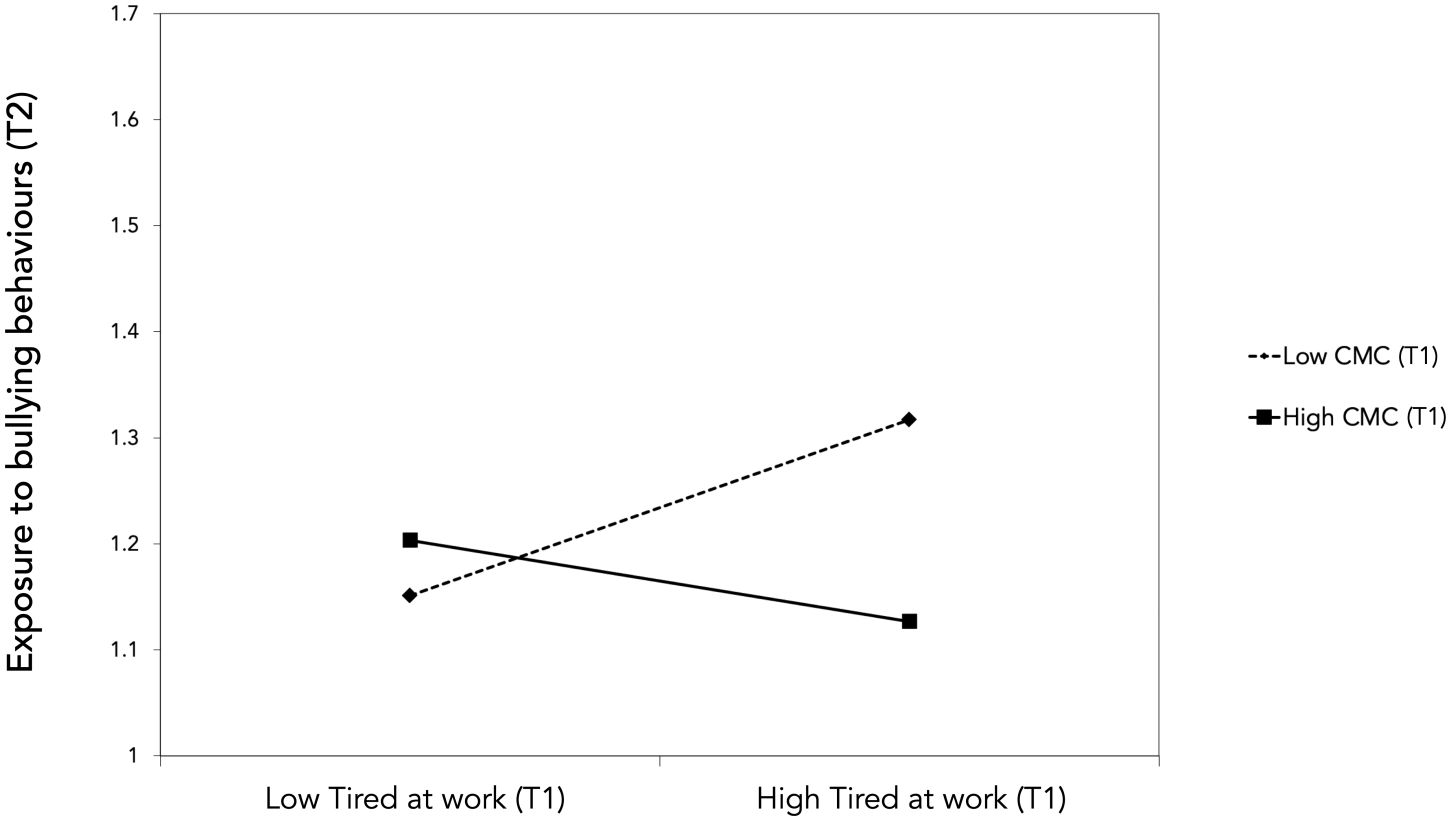
The interaction between tiredness at work and conflict management climate at T1 with regard to exposure to bullying behaviors at T2. High and low for the moderator and independent variable are +/− 1 SD from the mean

We hypothesized that CMC would be a moderator for the associations between exposure to bullying behavior and TAW, both for the forward and for the reversed association. The results are presented in Table 3. The reversed model (M6) provided the best fit, thus showing that CMC was a significant moderator of the association between TAW at baseline and exposure to bullying behaviors at follow‐up, β = −0.13, 95% CI [−0.19, −0.08]. To further test the reversed model (M6), we tested a model based on M6 that included the reciprocal association for CMC and exposure to bullying behaviors, χ^2^(550) = 1460.14, *P <* .001, CFI = 0.94, TLI = 0.93, RMSEA = 0.042 (90% CI = 0.040–0.045) and compared that with M6. The addition of the association between exposure to bullying behaviors at T1 and CMC at T2 improved the fit significantly, χ^2^(1) = 6.26, *P =* .012, see Figure 2. As displayed in Figure 3, TAW was positively associated with subsequent exposure to bullying among respondents who reported a weak CMC (−1 SD), b = 0.05, *P <* .001, whereas tiredness was negatively associated with subsequent exposure to bullying behaviors among targets who reported a strong CMC. However, at +1 SD from the mean of CMC it was not significant, b = −0.03, *P =* .090 (the association was significant at +1.16 SD from the mean of CMC). The results only support Hypothesis 4, not Hypothesis 3.

**TABLE 3:** Results of three cross‐lagged structural regression models between workplace bullying and tiredness at work including conflict management climate as moderator

		Test statistics					AIC
		χ^2^	*df*	CFI	TLI	RMSEA (90% CI)	
M5	Forward model (bullying T1 → tiredness T2)	1917.29***	551	0.91	0.90	0.052 (0.049–0.054)	78964.14
M6	Reversed model (tiredness T1 → bullying T2)	1466.40***	551	0.94	0.93	0.042 (0.040–0.045)	78830.98
M7	Reciprocal model	1954.01***	580	0.91	0.90	0.051 (0.048–0.053)	80901.18

Abbreviation

AIC, Akaike’s information criterion; CI, confidence interval; CFI, comparative fit index; RMSEA, root mean square error of approximation; TLI, Tucker–Lewis index.

*** *P < *.001.

## 4 DISCUSSION

This study investigated the bidirectional associations between TAW and exposure to bullying behaviors. The results showed a cross‐lagged effect from tiredness to subsequent bullying, but not the other way around, from exposure to bullying behaviors to feeling tired at work at follow‐up. This is the opposite results compared with the study by Nielsen and colleagues^20^ who found a cross‐lagged association between workplace bullying at T1 and insomnia at T2. As the study by Nielsen et al. is the only other cross‐lagged study on workplace bullying and sleep in existence, we will use this as the main comparison for the results of the current study. There are two major differences between the studies that could help understand the different results—the measure of sleep problems and the time‐lag. First, the measures of sleep problems and TAW are not the same. While Nielsen et al. focused on sleep problems in the form of insomnia, the current study measured aspects more directly connected to a possible consequence of poor sleep for one’s work. This could explain why the reversed model had the best fit to the data with a significant cross‐lagged effect from tiredness to subsequent exposure to bullying behaviors. Being tired at work may affect one’s performance.^2^, ^9^, ^19^ Reduced performance may trigger negative reactions from co‐workers and ultimately a negative treatment such as workplace bullying. A tired co‐worker may be irritable and easily provoked violating expectations of social conduct which may backfire making others return the frustration and possible aggression.^18^ Part of the explanation could also be that when feeling tired one has lower tolerance for misconduct and may, therefore, interpret others’ behaviors in a more negative light. Another explanation for the divergent findings to Nielsen and colleagues’ study^20^ is that the consequences of having problems sleeping may vary depending on other factors such as having a stimulating job. When measuring possible consequences of sleep problems, such as TAW, the link to subsequent bullying is likely to become clearer.

Another important explanation for the differences between this study and the study by Nielsen and colleagues^20^ is the time‐lag, 6 months compared with 18 months. Nielsen et al. suggested that the association between bullying and subsequent insomnia would be most noticeable in a shorter time span. Looking at this from a vicious circle perspective, sleep problems and TAW as a result of exposure to bullying behaviors may be something that can be discerned early on, however, as workplace bullying is an escalating process,^12^ the full impact from bullying may take longer to develop. The onset of different negative behaviors aimed at the target also go from more indirect to more direct acts.^37^ This may make it harder for the exposed to understand what is happening, leading to rumination and worrying affecting sleep early on. With the longer time‐lag in the present study the cross‐lagged effect from exposure to bullying behaviors to the related concept of tiredness was not significant in any of the models it occurred in (M2 and M4). When more time has passed, and the exposure to bullying behaviors continues it is no longer anything unexpected to the victim. In this case, feeling tired at work could be a minor issue, and other, more serious consequences of bullying, such as mental health problems could weaken the issue of mere tiredness 18 months later.

CMC emerged as a significant moderator for the reversed association between tiredness and exposure to bullying behaviors. When the CMC is poor, the impact of possible rule violations and difficulties to live up to expectations for a tired employee may hit with full force. If conflict management is lacking, interpersonal conflicts borne from frustration and aggression toward a tired co‐worker may escalate and become bullying if prolonged.^22^ A good CMC may on the other hand buffer possible conflicts that come out of this, lessen the impact,^28^, ^29^ or even reduces the occurrence as has been shown for bullying in general.^24^, ^25^, ^26^ Interesting to note is that the direct association between tiredness and bullying no longer was significant when adding CMC as a moderator. There were direct effects of CMC on exposure to bullying behaviors at both T1 and T2. The additional reciprocal association between exposure to bullying behaviors and CMC is an interesting find that point to the importance to deal with potential bullying situations early on as bullying has a negative effect on the trust in subsequent conflict management. As has been shown in previous studies, a good CMC is likely to reduce the occurrence of bullying behaviors since emerging conflicts may be dealt with before they are allowed to escalate and possibly becoming bullying.^27^

### 4.1 Strengths and limitations

The current study has several important strengths. It is based on a national probability sample of the whole Swedish workforce, it is a prospective study using time‐lagged data, and we have investigated the cross‐lagged associations of tiredness on subsequent exposure to bullying behaviors and vice versa. However, it should be noted that the use of a cross‐lagged design in itself prevents large cross‐lagged effects due to the stability in the model.^38^ A limitation is that the study is based on self‐report data, which may result in common method bias,^39^ however, using a time‐lag of 18 months between independent and dependent variables reduces this risk. The use of an 18‐month time‐lag also correspond to meta‐analytic findings on the stressor–strain effects over time as well as for the reversed effect.^21^ Ford et al. found that lagged effects tended to increase up until about three years. Comparing the current results to the study by Nielsen and colleagues^20^ indicates that the time‐lag for the forward and reversed effects for sleeping problems and bullying may need to be different to be able to capture the full effect of the reciprocal association. This is, however, a methodological problem hard to overcome. Combining the results from the two studies gives us a better understanding of the associations between sleep, tiredness, and bullying. The use of CMC on an individual level is also something worth discussing. CMC is a climate variable based on an estimate of the workplace as a whole. Ideally it would be treated as a group level variable, however, the nature of the current probability sample prevents that as each participant is randomly sampled from the whole Swedish workforce.

## 5 CONCLUSIONS AND IMPLICATIONS

The study results have important practical implications for employees, employers, and human resources personnel. As workplace tiredness has been established as a potential risk factor for being bullied at the workplace, an up‐front implication concerns the importance of finding ways to help employees to reduce tiredness, for example, by finding ways to improve sleep for employees not only will help them perform better at work, but also reduces the risk of them becoming targets of bullying. Regarding prevention, we have shown that developing a strong CMC is an effective way to counteract the effects of tiredness. As our results show, establishing a strong CMC can prevent bullying from occurring in the first place, and act as a buffer if a tired person provoke aggression from co‐workers. To build such a climate, it is imperative that supervisors and senior management in an organization: (a) show support and commitment through involvement and that they take quick and decisive action to correct problems or issues that may have a negative effect on interpersonal relations and conflict; (b) communicate with employees about issues that may affect safety and security, and bring these issues to the attention of the employees to create trust; (c) involve stakeholders including employees, unions, and health and safety representatives in the occupational health and safety process, through participation and consultation.^40^

## DISCLOSURE

*Ethical approval*: The project was approved by the Regional Ethical Review Board at Linköping University, Sweden. Protocol number: #2017/336‐32. *Informed consent*: All respondents received information about the study, its use, and opportunities to withdraw for an informed consent to participate. *Registry and the Registration No*. *of the study*/*Trial*: N/A. *Animal Studies*: N/A *Conflict of Interest*: The authors declare no Conflict of Interests for this article.

## AUTHOR CONTRIBUTIONS

M.R. and M.B.N. conceived the ideas; M.R. collected the data; M.R. analyzed the data; M.R. and M.B.N. wrote the manuscript.

## Data Availability

The data that support the findings of this study are available from the corresponding author upon reasonable request.
